# Iodine Map Radiomics in Breast Cancer: Prediction of Metastatic Status

**DOI:** 10.3390/cancers13102431

**Published:** 2021-05-18

**Authors:** Lukas Lenga, Simon Bernatz, Simon S. Martin, Christian Booz, Christine Solbach, Rotraud Mulert-Ernst, Thomas J. Vogl, Doris Leithner

**Affiliations:** 1 Department of Diagnostic and Interventional Radiology, University Hospital Frankfurt, 60590 Frankfurt, Germany; lukas.lenga@kgu.de (L.L.); simon.bernatz@kgu.de (S.B.); simon.martin@kgu.de (S.S.M.); christian.booz@kgu.de (C.B.); Rotraud.Mulert-Ernst@kgu.de (R.M.-E.); t.vogl@em.uni-frankfurt.de (T.J.V.); 2 Department of Gynecology and Obstetrics, University Hospital Frankfurt, 60590 Frankfurt, Germany; Christine.Solbach@kgu.de; 3 Department of Radiology, Memorial Sloan Kettering Cancer Center, New York, NY 10065, USA

**Keywords:** computed tomography, breast cancer, radiomics, dual-energy

## Abstract

**Simple Summary:**

Early and accurate diagnosis of breast cancer that has spread to other organs and tissues is crucial, as therapeutic decisions and outcome expectations might change. Computed tomography (CT) is often used to detect breast cancer’s spread, but this method has its weaknesses. The computer-assisted technique “radiomics” extracts grey-level patterns, so-called radiomic features, from medical images, which may reflect underlying biological processes. Our retrospective study therefore evaluated whether breast cancer spread can be predicted by radiomic features derived from iodine maps, an application on a new generation of CT scanners visualizing tissue blood flow. Based on 77 patients with newly diagnosed breast cancer, we found that this approach might indeed predict cancer spread to other organs/tissues. In the future, radiomics may serve as an additional tool for cancer detection and risk assessment.

**Abstract:**

Dual-energy CT (DECT) iodine maps enable quantification of iodine concentrations as a marker for tissue vascularization. We investigated whether iodine map radiomic features derived from staging DECT enable prediction of breast cancer metastatic status, and whether textural differences exist between primary breast cancers and metastases. Seventy-seven treatment-naïve patients with biopsy-proven breast cancers were included retrospectively (41 non-metastatic, 36 metastatic). Radiomic features including first-, second-, and higher-order metrics as well as shape descriptors were extracted from volumes of interest on iodine maps. Following principal component analysis, a multilayer perceptron artificial neural network (MLP-NN) was used for classification (70% of cases for training, 30% validation). Histopathology served as reference standard. MLP-NN predicted metastatic status with AUCs of up to 0.94, and accuracies of up to 92.6 in the training and 82.6 in the validation datasets. The separation of primary tumor and metastatic tissue yielded AUCs of up to 0.87, with accuracies of up to 82.8 in the training, and 85.7 in the validation dataset. DECT iodine map-based radiomic signatures may therefore predict metastatic status in breast cancer patients. In addition, microstructural differences between primary and metastatic breast cancer tissue may be reflected by differences in DECT radiomic features.

## 1. Introduction

Only recently, breast cancer has surpassed lung cancer as the most common type of malignancy worldwide, while it remains the leading cause of cancer death among women [1]. The development of breast cancer metastases is highly associated with unfavorable clinical prognosis, hence, early and reliable diagnosis of metastatic disease is essential to guide treatment decisions and improve patient outcome [2]. Computed tomography (CT) is used frequently in clinical practice for staging of breast cancer at risk of metastatic spread due to its high specificity and widespread availability. Nevertheless, CT mainly provides morphologic information, and has a moderate sensitivity for bone and visceral metastases, which may be detected in late stages or missed completely [3,4]. Alternative approaches to detect distant metastases such as 18F-fluorodeoxyglucose (FDG) positron emission tomography (PET)/CT also seem limited, as not all breast cancers display substantially increased glucose metabolism [5,6]. Thus, there is a strong need to develop reliable, quantifiable parameters to help predict metastatic status in breast cancer patients.

In recent years, dual-energy CT (DECT) has been increasingly implemented into clinical routines, providing a wide range of additional applications, such as iodine-selective imaging to quantify iodine concentrations as a marker for tissue vascularization. In parallel, radiomics, a computer-assisted image analysis technique which extracts and quantifies mathematical patterns from grey-level values of diagnostic medical images, has gained popularity within the imaging community [7,8]. These so-called radiomic features have been suggested to reflect underlying molecular processes, and may be linked to cancer characteristics such as proliferation and aggressiveness [9,10]. Coupled with artificial intelligence, as well as clinical and genomic data, radiomics has been evaluated as a potential adjunct tool in oncologic imaging, with good performance regarding the prediction of e.g., cancer subtype, treatment response and clinical outcome [11,12,13]. The value of radiomics signatures obtained from DECT iodine maps for breast cancer assessment has so far not been investigated, despite their informative value on tumor biology potentially being of interest [14].

Therefore, the goal of this study was to investigate whether radiomic signatures derived directly from DECT iodine maps facilitate the prediction of breast cancer metastatic status, and whether there are textural differences between primary breast cancers and metastases. We hypothesized that variations in metastatic potential between different breast cancers would lead to microscopical alterations in tumor perfusion heterogeneity, which could be captured by iodine map-based radiomic features.

## 2. Results

Seventy-seven treatment-naïve patients with biopsy-proven breast cancers met our criteria for enrollment in the study: 41 cancers were non-metastatic (53.2%) and 36 metastatic (46.8%), with metastases in the following locations: axillary lymph nodes n = 35; liver, n = 5; bone, n = 6; lung, n = 4; soft tissue, n = 2. Only 10 patients showed distant metastases, some of which, however, had more than one metastatic site, with a combined total of 17 distant metastatic sites. None of the patients in the non-metastatic group had developed metastatic disease at a later time point (mean follow-up time 13.1 ± 5 months).

In five patients, metastatic disease could not be identified on CT as such, but was confirmed via histopathological analysis. In these cases, axillary lymph node metastases were found in surgical specimens; the histopathological report described micrometastases which were not visible on CT. Distant metastases were not present in these patients. The underlying breast cancer biology in this group was as follows: luminal A, n = 4; triple negative, n = 1.

Mean breast lesion size was 30 ± 19.1 mm (range, 9–100 mm); mean patient age was 54.8 ± 14.7 years (range, 27–88 years). Sixty-nine cancers were invasive ductal carcinomas, while 8 were invasive lobular carcinomas; 53 were HR positive (68.8%). Forty-two cancers were classified as luminal A (54.5%), 11 as luminal B (14.3%), 6 as HER2-enriched (7.8%), and 18 as triple negative (23.4%). Chi-Square test revealed no significant difference (*p* = 0.79) in distribution of molecular subtypes between the metastatic (19 luminal A, 5 luminal B, 4 HER2-enriched, 8 triple negative) and non-metastatic group (23 luminal A, 6 luminal B, 2 HER2-enriched, 10 triple negative). Two patients had grade 1 (2.6%), 44 grade 2 (57.1%), 31 grade 3 breast cancers (40.3%). Differences in tumor grade distribution between metastasized (22 grade 2, 14 grade 3) and non-metastasized patients (2 grade 1, 22 grade 2, 17 grade 3) were also not significant (*p* = 0.373).

**Figure 1:**
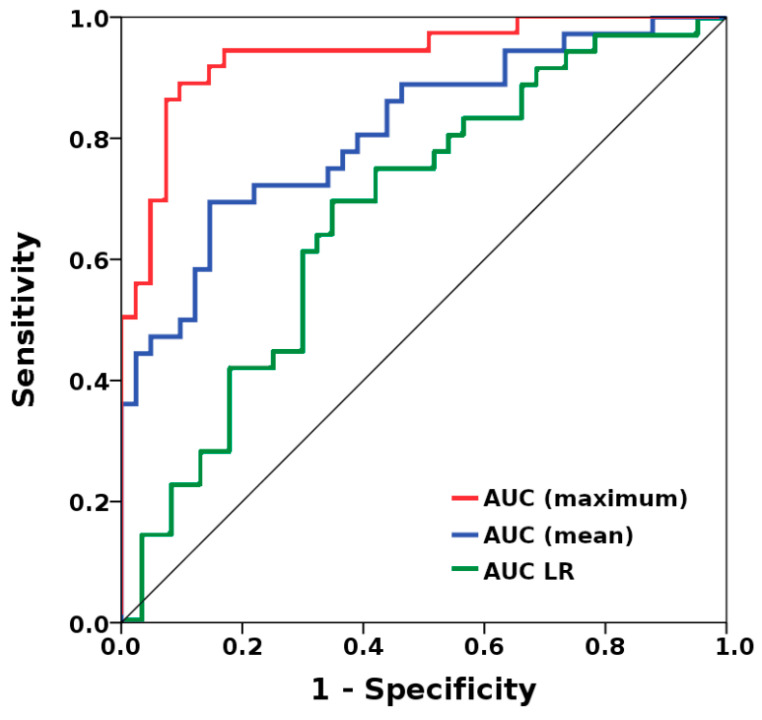
Multilayer perceptron neural network (MLP-NN)-based separation of metastatic and non-metastatic breast cancers yielded a maximum area under the receiver operating characteristic (ROC) curve (AUC) of 0.94, and a mean AUC of 0.82, while logistic regression (LR)-based separation yielded an AUC of 0.69.

MLP-NN-based prediction of metastatic status yielded good results, with AUCs of up to 0.94, and accuracies of up to 92.6 in the training and 82.6 in the validation datasets. Logistic regression (LR)-based prediction of metastatic status yielded an AUC of 0.69, with an accuracy of 59.7% for the entire cohort (Figure 1). Additional data are provided below.

Discrimination of primary tumor and metastatic tissue was substantial with AUCs of up to 0.87, and accuracies of up to 82.8 in the training and 85.7 in the validation datasets. Additional results are given in Table 1.

## 3. Discussion

This is the first study to evaluate the utility of radiomic signatures derived from DECT iodine maps to predict breast cancer metastatic status. Our results suggest that iodine map radiomic features carry potentially useful information for the noninvasive prediction of the presence of metastases, as well as on tumor biology with regard to differences between primary and metastatic cancer tissue.

In breast cancer, wide variations in metastatic potential exist even when molecular subtype, tumor size, stage and grade are identical/comparable [2]. In the setting of advanced breast cancer, guidelines stipulate that primary tumor assessment should always be complemented by whole-body imaging. However, there is still discordance with regard to the whole-body imaging test of choice, as each technique has its weaknesses [15]. Also, conventional imaging biomarkers such as HU and SUVmax to predict metastatic disease have so far proven unsatisfactory or have not been sufficiently validated [16,17]. In this setting, radiomics may offer a unique opportunity to oncologic imaging, yielding several parameters of the whole, potentially heterogeneous tumor by means of post-processing, which is neither cost- nor time-intensive. Previous studies investigating the potential of radiomic signatures obtained from different imaging techniques to predict metastatic disease in breast cancer focused exclusively on locoregionary axillary lymph node metastases, yielding good to excellent results [18,19]. For instance, Zheng et al. demonstrated an AUC of 0.90 for the discrimination between axillary breast cancer metastases and disease-free axilla based on radiomic features from ultrasound and sheer wave elastography, as well as clinical parameters [20].

**Figure 2:**
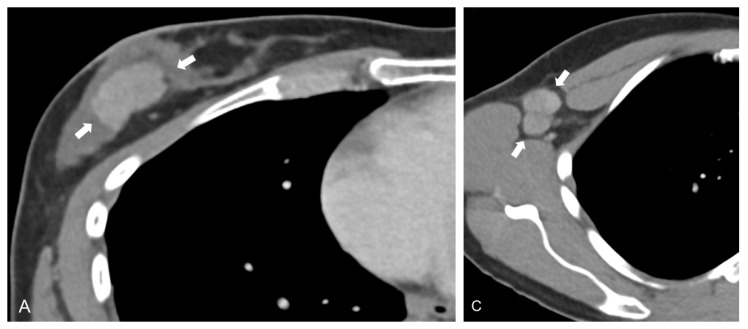

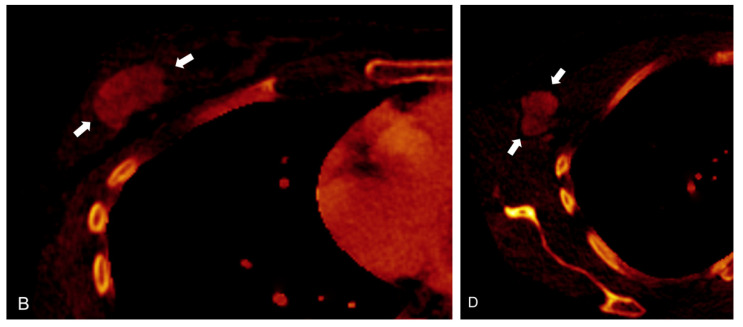
Axial contrast-enhanced dual-energy CT (DECT) scan of a 40-year-old patient with grade 2 luminal B invasive ductal carcinoma in the right breast. Linearly blended M_0.6 image series show a lesion in the right breast (**A**), as well as enlarged, round lymph nodes in the right axilla (**B**). In the present study, DECT iodine map radiomic signatures derived from the primary tumor (**C**) yield a mean AUC of 0.82 for separation of metastatic and non-metastatic breast cancers; in addition, substantial textural differences exist between primary tumor and metastatic tissue (**D**).

While CT-derived radiomic signatures have been evaluated in other body regions to predict distant metastases, e.g., in lung adenocarcinoma by Coroller et al. [21], they have not been used in breast cancer for this particular purpose, but instead, for prediction of treatment response [22]. Despite their selective quantification and visualization of tumor blood supply, data is particularly scarce on the significance of DECT iodine map radiomic features in oncologic imaging. In one of the few available studies, Zhou et al. demonstrated excellent performance of iodine map radiomic combined with CT imaging features in diagnosing cervical lymph node metastases of thyroid cancer, yielding an AUC of 0.895 in the validation dataset [23]. While their model was based on lymph node feature extraction, we stratified patients into metastatic and non-metastatic groups based on features of the treatment-naïve primary breast tumor (Figure 2).

Meanwhile, Choe et al. successfully predicted survival outcomes in 93 lung cancer patients using iodine overlay map radiomic features [24]. They identified histogram entropy as a single-feature predictor for survival, while in our model, principal components derived from the entire spectrum of radiomic feature classes were utilized for classification. This approach has been previously used in the literature [25], and, contrary to histogram-based features, also includes true descriptors of spatial signal heterogeneity. Iodine map heterogeneity can be assumed to differ from heterogeneity of unenhanced and contrast-enhanced CT images, as it selectively depicts tissue perfusion and permeability, which are linked to tumor angiogenesis and, hence, aggressiveness [26]. Aggressiveness, in turn, is linked to the development of metastases, and therefore, radiomic features of DECT iodine-selective images may carry predictive information. If validated externally, we hypothesize that DECT radiomic signatures might even serve as prognostic markers, although this topic was not specifically investigated in our present study.

In addition to the above, we investigated whether microstructural and biologic differences that are known to exist between primary tumors and metastases [27] would also be reflected by differences in iodine map texture features. Our findings support this assumption: radiomic features enabled differentiation between primary breast cancers and their metastases, with AUCs of up to 0.87. These findings are in good accordance with radiomics studies in other malignancies, such as lung cancer [28,29]. These results might be relevant in situations where more than one primary tumor exists and it is unclear from which tumor the metastasis arises.

This study has limitations beyond its retrospective design and the moderate cohort size. First, the number of radiomic features considered in our analysis was limited—for example, wavelet transformation was not used as a pre-processing step, which would have doubled the number of features. However, in view of our sample size, a substantial increase in calculated radiomic features would have clearly increased the risk of overfitting [7,30]. The same is true for our choice of classification algorithm. While MLP-NN is a well-established machine learning technique, more advanced algorithms are available, but would probably have been too complex for use in our cohort size, again making the model prone to overfitting. Third, a true prognostic part of our study is missing, as metastatic status is assessed using the presence of metastases at baseline staging DECT, and cancers may metastasize in the future. To address this issue, we followed up on patients without metastases at first presentation to find that none of these patients developed metastatic disease at a later time point (mean follow-up 13.1 ± 5 months). While we did not formally investigate the value of iodine map radiomics for survival prognostication, the fact that none of the patients within the non-metastatic group developed metastases might possibly indicate a prognostic value. However, no threshold calculation was performed, since the lack of metastases in the non-metastatic group was observed in retrospect. Survival prognostication was not attempted due to heterogeneity in terms of subsequent treatment in combination with the limited sample size. In addition, the duration of follow-up in patients without metastases is shorter than two years, and hence, limited. Future analyses are required to investigate the true prognostic value of iodine map radiomics in that regard. Fourth, of the 36 patients with metastases, 35 have axillary lymph node metastases. The presence or absence of axillary metastases can be diagnosed by other methods such as ultrasound echogram or fine needle aspiration cytology. Fifth, the subcohort of patients with distant metastases is clinically the most relevant, however, only a total of ten patients showed distant metastases. Given the very small size of this subcohort, there is concern that radiomics-based differentiation between patients with and without distant metastases could be subject to overfitting, i.e., producing overoptimistic estimates of classification performance (an intrinsic limitation of radiomics/machine learning studies). In addition, with only ten patients in the distant metastasis group, there is considerable class imbalance, which is another common pitfall in radiomics research and might limit the generalizability of our results [7]. Hence, we did not attempt this classification in the present study; further research in a (much) larger patient population is needed in that regard. Finally, iodine map measurements hold inherent limitations due to varying tissue enhancement caused by either contrast agent infusion parameters such as volume and flow rate, or patient features such as blood pool and cardiac output. Further investigations are needed to demonstrate meaningful, robust use of radiomic analysis based on iodine-selective imaging, as well as to evaluate a potential link between the prediction of metastases by iodine map radiomics and tumor biology examined by multigene assays such as MammaPrint.

## 4. Materials and Methods

### 4.1. Patients and Design

This retrospective single-center study was approved by the local Institutional Review Board with a waiver for written informed consent. Our Picture Archiving and Communication System and Radiology Information System was searched by a board-certified radiologist with six years of experience to identify patients who underwent contrast-enhanced venous phase DECT chest, abdomen, pelvis for baseline staging of newly diagnosed breast cancer between January 2017 and February 2019. In our institution, CT-based M-staging of breast cancer is performed in the setting of high risk of disease spread, such as stage T3/4 cancers, higher-grade cancers, high Ki-67, and, to some extent, multifocal and multicentric disease [31]. All patients met the following inclusion criteria: histopathologically verified treatment-naïve breast cancer; patient age 18 years or older. Exclusion criteria were: breast cancer types other than invasive ductal carcinoma and invasive lobular carcinoma; history of other (non-breast) cancers; history of local or systemic cancer treatment; and primary tumor not visible on CT imaging. Thus, based on these criteria, a total of 77 consecutive patients were included in this study.

Histopathological analyses of surgical specimens and biopsy samples have been performed on all retrospective patients. All patients without distant metastases after initial staging examinations have been treated with either breast-conserving therapy or mastectomy. Patients with suspected distant metastases on CT received CT- (n = 13) or ultrasound-guided (n = 4) diagnostic biopsy of the liver, bone, lung, and soft tissue. Biopsies of the primary breast lesion have been performed in all patients. Tumor histology, grade and immunohistochemical status including estrogen receptor, progesterone receptor, and HER2 were obtained from histopathological reports by reference pathologists based on analysis of surgical specimens [32]. Subsequent histopathological reports of patients without metastases on baseline DECT were reviewed with regard to development of metastatic disease at a later time point.

### 4.2. CT Imaging and Post-Processing

All patients were examined using a 192-slice third-generation dual-source CT scanner (Somatom Force, Siemens Healthineers, Forchheim, Germany) in dual-energy mode (tube A 90 kV/95 mAs, tube B 150 kV/59 mAs with tin filtration). All examinations were performed using automatic tube current modulation (Caredose 4D, Siemens Healthineers). Non-ionic iodinated contrast media (Imeron 400, Bracco, Milan, Italy) adjusted to patient weight (1.2 mL/kg body weight) was administered via an antecubital vein at a flow rate of 2–3 mL/s. Venous phase image data were acquired after a fixed scan delay of 70 s in craniocaudal direction with a pitch of 0.7, and collimation of 2 × 192 × 0.6 mm. Axial, coronal and sagittal image series were reconstructed with a matrix size of 512 × 512, thickness of 3 mm and increment of 1.5. Advanced modelled iterative reconstruction algorithm (Admire, Siemens Healthineers) was applied at a strength level of 3 out of 5. Iodine maps were generated consistently in axial sections with 3 mm thickness and 1.5 mm interslice gap using dedicated software at 100% (syngo.via, Version VB10B, Siemens Healthineers) [33].

### 4.3. Radiomics Analysis

**Figure 3:**
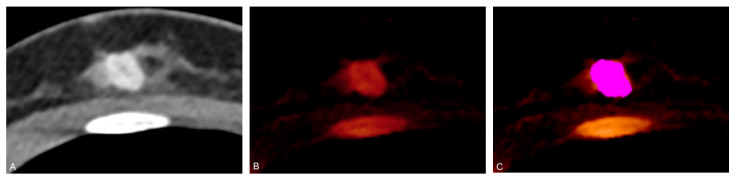
Axial contrast-enhanced dual-energy CT (DECT) scan of a 47-year-old woman with grade 1 luminal A invasive ductal carcinoma in the right breast. Linearly blended M_0.6 series (**A**) demonstrate a contrast-enhancing mass; pure iodine maps (**B**) reconstructed from DECT datasets display iodine content within the lesion. A three-dimensional volume of interest (VOI) (**C**) is placed semi-automatically on the iodine map for radiomic analysis.

Feature extraction was performed semi-automatically using the open-source software LIFEx 6.0 (https://lifexsoft.org/ (accessed on 28 January 2021)) [34]. Two board-certified radiologists with six and seven years of experience analyzed all images in consensus. Primary breast tumors and, separately, metastases were identified using M_0.6 linearly blended series (combining 60% low, 40% high tube voltage spectrum) and iodine maps in accordance with histopathological reports. Iodine maps only were used for radiomics analysis. Three-dimensional volumes of interest (VOI) covering the entire breast tumor, and, if present separately the metastatic volume, were constructed semi-automatically on axial images: in a first step a threshold interval of 40–500 HU was used for automatic segmentation, which in a second step was validated, and, if necessary, adjusted by a board-certified radiologist (Figure 3). Multicentric, multifocal, or bilateral disease was included in the feature extraction. Adjacent carcinoma in situ components, biopsy markers and artefacts were excluded from segmentation.

Radiomic features were derived from various feature classes: first-order histogram (n = 5), which represent statistical descriptors of signal intensities; shape-derived (n = 5), describing sphericity, compacity and volume of tumors; conventional and discretized indices (n = 7), which give quartiles of data; and more sophisticated second order metrics, such as grey-level co-occurrence matrix (GLCM; n = 6), which are based on pairs of voxels and provide information on lesion heterogeneity; grey-level run-length matrix (GLRLM; n = 11), giving the size of homogeneous runs for each grey-level in four directions; neighborhood grey-level difference matrix (NGLDM; n = 3), which describe grey-level intensity differences between a single voxel and its neighboring voxels in three dimensions; grey-level zone length matrix (GLZLM; n = 11), measuring the size of homogeneous grey-level zones in three dimensions; resulting in a total number of 48 features per breast cancer lesion [34]. Contrary to several other software packages, LIFEx calculates mean values of features for different orientations or interpixel distances, e.g., entropy (1, 1), entropy (1, −1), etc. To yield radiomics signatures of metastatic volume, all metastases were considered together; therefore, shape-derived features were not taken into account, resulting in a total of 43 features per combined metastatic volume. Cases where metastatic disease was not visible on CT, but detected by another imaging test and confirmed by histopathological analysis, were assigned to the metastatic group.

### 4.4. Statistical Analysis

Following feature extraction, principal component analysis (PCA) was used for dimensionality reduction, i.e., to decrease the degree of information redundancy due to correlations between individual features and hence, reduce the risk of overfitting [30]. Out of the many features obtained, PCA calculated principal components based on Eigenvalues >1, constituting the radiomic signature. For subsequent classification, the principal radiomic components were fed into a multilayer perceptron, feed-forward, artificial neural network (MLP-NN), which is based on a backpropagation algorithm [35]. MLP-NN was used to separate (1) patients with metastases from non-metastatic patients, as well as (2) primary breast cancer lesions from metastatic tissue. For each pairwise comparison, 70% of cases were randomly assigned to the training, and 30% to the validation dataset. MLP-NN relies on the probability of class membership, based on a combination of weighted input variables rather than on a simple single-feature threshold value, to determine if a case is rated as positive or negative. The utilized activation function (softmax) assigns each case to the class (i.e., metastatic or non-metastatic) with the higher probability value, i.e., based on a >0.5 probability threshold. As MLP-NN starts at an initial guess at the weights of each principal radiomic component, each classification step was performed 40 times each. The neural network operated at a minimum of one hidden layer and a minimum of two neurons per hidden layer. Areas under the ROC curves (AUCs) and classification accuracies for training and validation datasets were calculated. For comparative purposes, LR with forward selection was performed in addition to MLP-NN for separation of metastatic and non-metastatic primary breast cancers.

Chi-square test was applied to examine differences in distribution of molecular subtypes and tumor grades between the metastatic and non-metastatic group. All statistical analyses were performed using IBM SPSS 24.0 (IBM Corp., Armonk, NY, USA). A *p*-value below 0.05 was considered statistically significant.

## 5. Conclusions

In conclusion, our results suggest that DECT iodine map-derived radiomic signatures have the potential to predict metastatic status in breast cancer patients. In addition, microstructural differences between primary and metastatic breast cancer tissue are also reflected by differences in the respective DECT radiomic features. In patients at high risk of metastatic spread based on radiomic analysis but without visible metastases on CT, another imaging modality such as PET or shorter follow-ups could be indicated. Larger prospective studies using advanced neural networks are warranted to confirm our initial findings and fully clarify the predictive potential of radiomics in this context.

## Figures and Tables

**Table 1:** Classification AUCs and accuracies for radiomics data.

	Mean	Median	IQR	Range
**Metastatic vs. non-metastatic breast cancers:**				
AUC	0.82	0.81	0.78–0.84	0.77–0.94
Accuracy training (%)	75.78	75.9	74.1–76.83	66.7–92.6
Accuracy validation (%)	73.92	73.9	69.6–78.3	65.2–82.6
**Primary breast cancers vs. metastases:**				
AUC	0.81	0.81	0.80–0.83	0.79–0.87
Accuracy training (%)	74.87	74.75	72.8–77.08	61.5–82.8
Accuracy validation (%)	72.87	73.2	69–77.8	56–85.7

Note: AUC, area under the curve; IQR, interquartile range.

## Data Availability

The data presented in this study are available on request from the corresponding author. The data are not publicly available due to data protection policies.
